# New Furanones from the Plant Endophytic Fungus *Pestalotiopsis besseyi*

**DOI:** 10.3390/molecules171214015

**Published:** 2012-11-27

**Authors:** Haitao Liu, Shuchun Liu, Liangdong Guo, Yonggang Zhang, Langjun Cui, Gang Ding

**Affiliations:** 1 Key Laboratory of Bioactive Substances and Resources Utilization of Chinese Herbal Medicine, Ministry of Education, Institute of Medicinal Plant Development, Chinese Academy of Medical Sciences and Peking Union Medical College, Beijing 100193, China; 2 Graduate School of Chinese Academy of Sciences, Beijing 100086, China; 3 Institute of Microbiology, Beijing 100086, China; 4 Biotechnology Center of Shandong Academy of Sciences, Jinan 250014, Shandong, China; 5 College of Life Science, Shaanxi Normal University, Xi’an 710062, Shaanxi, China

**Keywords:** pestalafuranones A–E, endophytes, *Pestalotiopsis besseyi*, bioactivity

## Abstract

Pestalafuranones A–E (compounds **1**–**5**), five new 2(*5H*)-furanones, have been isolated from cultures of an isolate of *Pestalotiopsis besseyi*. The structures of these compounds were elucidated mainly by analysis of their NMR spectroscopic data and HRESIMS experiments. Pestalafuranones A–C (compounds **1**–**3**) displayed weak inhibitory effects against HIV-1 replication in C8166 cells, whereas pestalafuranones D (**4**) and E (**5**) showed moderate antifungal activity against the plant pathogens *Verticillium dahiae* and *Alternaria longipes*.

## 1. Introduction

Plant endophytic fungi have been demonstrated to be rich sources of bioactive secondary metabolites [1,2], and *Pestalotiopsis* spp. are especially well-known as producers of bioactive natural products [3,4,5,6]. In the course of our search for new bioactive secondary metabolites from *Pestalotiopsis* spp., an isolate of *P. besseyi* (1009) which was grown in a solid-substrate fermentation culture was investigated. The organic solvent extract displayed inhibitory effect on HIV-1 replication in C8166 cells, and antifungal activity against the plant pathogens *Verticillium dahiae* (CGMCC 3758) and *Alternaria longipes* (CGMCC 2875). Bioassay-guided fractionation of this extract led to the isolation of five new 2(*5H*)-furanones namely pestalafuranones A–E (compounds **1**–**5**). Detailed isolation, structure elucidation, and bioactivities of these compounds are presented herein.

## 2. Results and Discussion

### 2.1. Structural Elucidation

**Figure 1:**
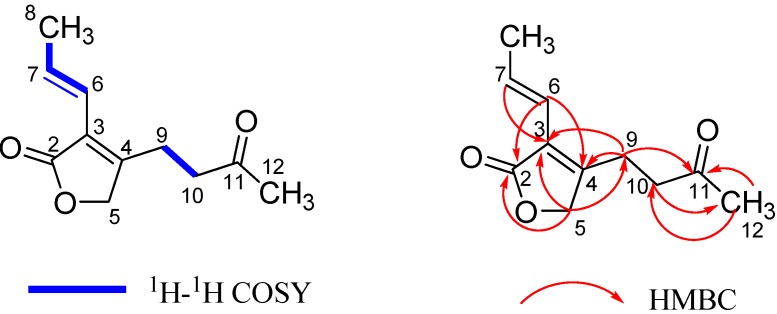
^1^H-^1^H COSY and Key HMBC correlations for pestalafuranone A (**1**).

**Figure 2:**
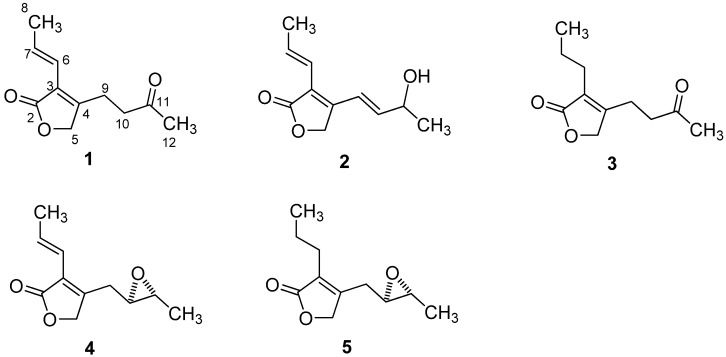
Structures of compounds **1**–**5**.

The molecular formula of pestalafuranone A (**1**) was established as C_11_H_14_O_3_ (five degrees of unsaturation) by analysis of its HRESIMS [*m/z* 217.0840 (M+Na)^+^; Δ +0.5 mmu] and NMR data (Table 1 and Table 2). Interpretation of the ^1^H-, ^13^C-, and HMQC-NMR spectroscopic data of **1** indicated the presence of two methyl groups, three methylene units (one oxygenated), one disubstituted and one tetrasubstituted olefin, and two carbonyl carbons, leaving one degree of unsaturation and this suggesting **1** to be a monocyclic compound. Analysis of the ^1^H–^1^H COSY NMR data led to the identification of two isolated proton spin-systems corresponding to the C-6–C-8 and C-9–C-10 subunits of structure **1**. HMBC correlations from H-7 to C-3 and from H-6 to C-2, C-3, and C-4 led to the connection of C-3 to C-6, whereas correlations from H_2_-10 to C-4, H_2_-9 to C-3, C-4, C-5, and from the oxygenated methylene protons H_2_-5 to C-3, C-4 and C-9 indicated that C-3, C-5, and C-9 were all connected to C-4. Key HMBC correlation of H_2_-5 with C-2 established the α,β-unsaturated γ-lactone subunit. Cross peaks from Me-12 to C-10, C-11 and from CH_2_-10 to C-11, C-12 in the HMBC spectra (Figure 1) confirmed that C-11 was connected to C-10 and C-12. Thus the structure of **1** was determined as shown in Figure 2.

**Table 1:** ^1^H-NMR Data for **1**–**3**, **5** in CDCl_3_ and **4** in acetone-*d*_6_.

Position	1 *^a^*	2 *^a^*	3 *^a^*	4 *^a^*	5 *^a^*
5	4.65, s	4.85, s	4.63, s	4.78, s	4.78, d (17)
					4.71, d (17)
6	6.10, d (16)	6.26, d (16)	2.45, t (7.5)	6.24, d (16)	2.61, t (7.5)
7	6.86, m	6.97, m	1.52, m	6.85, m	1.55, m
8	1.86, d (7.0)	1.89, d (6.7)	0.93, t (7.5)	1.81, d (6.5)	0.94, t (7.5)
9	2.70, m	6.77, d (16)	2.67, br s	2.90, dd (15, 3.6)	2.86, dd (12, 4.0)
				2.65, dd (15, 6.0)	2.44, dd (12, 7.0)
10	2.70, m	6.04, dd (16, 7.7)	2.67, br s	2.77, m	2.75, ddd (7.0, 4.0, 2.0)
11		4.52, m		2.85, dq (4.5, 2.0)	2.81, dq (7.5, 2.0)
11-OH		1.75, br s			
12	2.18, s	1.68, d (7.0)	2.19, s	1.24, d (4.5)	0.93, d (7.5)

*^a^* Recorded at 500 MHz. *J* values are given in Hz.

**Table 2:** ^13^C-NMR data for **1**–**3**, **5** in CDCl_3_ and **4** in acetone-*d*_6_.

Position	1 *^a^*		2 *^a^*		3 *^a^*		4 *^a^*		5 *^a^*
2	172.9, qC		173.0, qC		177.5, qC		173.0, qC		174.5, qC
3	123.3, qC		122.7, qC		127.9, qC		123.9, qC		128.7, qC
4	156.6, qC		149.4, qC		158.9, qC		156.3, qC		158.5, qC
5	70.8, CH_2_		68.6, CH_2_		71.2, CH_2_		71.7, CH_2_		71.8, CH_2_
6	118.1, CH		118.7, CH		25.6 CH_2_		119.9, CH		25.6, CH_2_
7	133.0, CH		134.3, CH		20.6 CH_2_		132.3, CH		21.4, CH_2_
8	19.3, CH_3_		19.6, CH_3_		13.9, CH_3_		17.4, CH_3_		13.9, CH_3_
9	20.4, CH_2_		118.6, CH		21.3, CH_2_		30.2, CH_2_		29.9 CH_2_
10	41.0, CH_2_		140.8, CH		41.3, CH_2_		57.2, CH		56.8, CH
11	206.1, qC		68.3, CH		206.0, qC		54.7, CH		54.5, CH
12	29.8, CH_3_		23.5, CH_3_		29.8, CH_3_		19.1, CH_3_		17.2, CH_3_

*^a^* Recorded at 125 MHz.

The molecular formula of pestalafuranone B (**2**) was determined to be C_11_H_14_O_3_ by analysis of its HRESIMS (*m/z* 217.0842 [M+Na]^+^; Δ +0.7 mmu) and NMR data (Table 1 and Table 2). Analysis of the ^1^H and ^13^C-NMR data of **2** revealed the presence of structural features similar to those found in **1**, except the signal for the ketone group (δ_C_ 206.1) present in **1** was replaced by an oxymethine (δ_H_ 4.52; δ_C_ 68.3), and the two methylene units CH_2_-9 and CH_2_-10 were replaced by two olefinic methines (δ_H_ 6.77 and 6.04). The ^1^H–^1^H COSY correlations for pestalafuranone B finally confirmed the above observations, leading to the assignment of the planar structure of **2**. 

Pestalafuranone C (**3**) was assigned a molecular formula of C_11_H_16_O_3_ (four degrees of unsaturation) on the basis of HRESIMS analysis [*m/z* 219.0998 (M+Na)^+^; Δ +0.6 mmu] and NMR data (Table 1 and Table 2), thus having two mass units more than **1**. Analysis of the ^1^H- and ^13^C-NMR data for **3** indicated that the C-6/C-7 olefinic bond observed in **1** was reduced to give two methylene units, which was further confirmed by analysis of its ^1^H–^1^H COSY NMR data. 

The molecular formula of pestalafuranone D (**4**) was established as C_11_H_14_O_3_ (five degrees of unsaturation) by analysis of its HRESIMS [*m/z* 217.0846 (M+Na)^+^; Δ +1.1 mmu] and NMR data (Table 1 and Table 2). The ^1^H- and ^13^C-NMR data of **4** suggested the presence of the same α,β-unsaturated γ-lactone subunit with a propenyl side chain attached to C-3, as that appearing in compounds **1** and **3**. Analysis of the ^1^H–^1^H COSY NMR data of **4** led to the identification of the C-9–C-12 partial structure. Considering the ^13^C chemical shifts for C-10 (δ_C_ 57.2) and C-11 (δ_C_ 54.7) as well as the unsaturation index, C-10 and C-11 must be attached to the remaining oxygen to form an epoxide moiety. Therefore, the planar structure of pestalafuranone D was elucidated as depicted in **4**.

The molecular formula of pestalafuranone E (**5**) was determined to be C_11_H_16_O_3_ on the basis of HRESIMS analysis [*m/z* 219.0995 (M+Na)^+^; Δ +0.3 mmu] and NMR data (Table 1 and Table 2). Examination of the ^1^H- and ^13^C-NMR data revealed the close resemblance of **5** to **4**, except for the reduction of the C-6/C-7 olefinic bond in **4** giving the structure of **5**, which was further supported by relevant ^1^H–^1^H COSY correlations. 

The C-6/C-7 double bond in compounds **1**, **2** and **4**, and the C-9/C-10 double bond in **2**, were assigned an *E*-geometry on the basis of the coupling constants observed for the corresponding olefinic protons (*J* = 16 Hz). The vicinal coupling constant of *J* = 2.0 Hz between H-10 and H-11 in compounds **4** and **5** suggested a *cis* relationship between these two protons [7]. Due to the limited amount available of compound **2**, its absolute configuration was not determined.

### 2.2. Anti-HIV Activity

Pestalafuranones A–E (compounds **1**–**5**) were tested for *in vivo* activity against HIV-1. Pestalafuranones A–C (compounds **1**–**3**) showed weak inhibitory effects on HIV-1 replication in C8166 cells, with EC_50_ values of 10.52, 24.32 and 36.74 μg/mL, respectively (the EC_50_ values of positive control zidovudine (AZT) and indinavir sulfate (IDV) were 2.17 ng/mL and 3.92 ng/mL, respectively). 

### 2.3. Antifungal Activity

In the antifungal bioassay, pestalafuranone D (**4**) showed weak activity against the plant pathogenic fungi *Verticillium dahiae* (CGMCC 3758) with IC_50_ values of 24.5 μg/mL, whereas pestalafuranone E (**5**) displayed moderate activity against *Alternaria longipes* (CGMCC 2875) with IC_50_ values of 10.3 μg/mL (positive control fluconazole showed IC_50_ value of 0.50 and 0.30 μg/mL). 

## 3. Experimental

### 3.1. General

Optical rotations were measured on a Perkin-Elmer 241 polarimeter, and UV data were recorded on Shimadzu Biospec-1601 spectrophotometer. IR data were recorded using a Nicolet Magna-IR 750 spectrophotometer. ^1^H- and ^13^C-NMR data were acquired with Varian Mercury-400/500 spectrometers using solvent signals (CDCl_3_; *δ*_H_ 7.26/*δ*_C_ 77.7; acetone-*d*_6_; *δ*_H_ 2.05/*δ*_C_ 29.8, 206.0) as references. The HMQC and HMBC experiments were optimized for 145.0 and 8.0 Hz, respectively. ESIMS data were recorded on a Bruker Esquire 3000^plus^ spectrometer. HRESI MS data were obtained using a Bruker APEX III 7.0 T spectrometer.

### 3.2. Fungal Material

The culture of *P. besseyi* was isolated by Professor Guo from Dongling Mountain, Beijing, in May 2005. The isolate was identified by Prof. Guo, and assigned the accession number 1,009 in his culture collection at the Institute of Microbiology, Chinese Academy of Sciences, Beijing. The isolate was subcultured on PDA slants at 25 °C for 15 days. The agar plugs were used to inoculate 250-mL Erlenmeyer flasks, each containing 50 mL of media (0.4% glucose, 1% malt extract, and 0.4% yeast extract), and the final pH of the media was adjusted to 6.5 before sterilization. Flask cultures were incubated at 25 °C on a rotary shaker at 170 rpm for five days. Twenty 500-mL Erlenmeyer flasks, each containing 150 mL of liquid media (6% dextrin, 2% maltose, 0.75% cotton-seed meal, 0.7% peptone, 0.25% CaCO3, 0.25% gSO4·7H2O, 0.1% FeSO4·7H2O, 0.001% ZnSO4; final pH 6.0) and 30 g of vermiculite were individually inoculated with 15 mL of the seed culture, and incubated at 25 °C for 10 days.

### 3.3. Extraction and Isolation

The fermented material was freeze-dried and extracted with methyl ethyl ketone (3 × 500 mL), and the organic solvent was evaporated to dryness under vacuum to afford 5.0 g of crude extract. The extract was fractionated by silica gel column chromatography (5 × 40 cm) using CHCl_3_–CH_3_OH gradient elution. The fraction (50 mg) that was eluted with 1% MeOH was further separated by semipreparative reversed-phase HPLC (Kramosil C_18_ column; 10-µm; 10 × 250 mm, 2 mL/min) to afford pestalafuranone B (**2**; 1.9 mg, *t*_R_ 15.3 min; 50% MeOH in H_2_O over 2 min, 50–72% over 10 min, 72–74% over 13 min). The fraction (180 mg) that was eluted with 2% MeOH was then separated by Sephadex LH-20 column chromatography (CH_2_Cl_2_–*n*-C_6_H_14_ = 4:1) to afford six fractions. Fraction 3 (60 mg) was successively separated by semipreparative reversed-phase HPLC (32% MeOH in H_2_O over 2 min, 32–55% over 60 min) afforded pestalafuranones A (**1**; 1.6 mg, *t*_R_ 25.7 min), C (**3**; 6.0 mg, *t*_R_ 26.6 min), D (**4**; 1.5 mg, *t*_R_ 30.0 min), and E (**5**; 3.5 mg, *t*_R_ 31.5 min).

*Pestalafuranone A* (**1**): colorless oil; UV (CH_3_OH) λ_max_ 272 (*ε* 7800), 265 (*ε* 6600) nm; IR (Neat) *ν*_max_ 2962, 1748, 1717, 1448, 1364 cm^−1^; ^1^H- and ^13^C-NMR data, see Table 1 and Table 2; HRESIMS obsd. *m/z* 217.0840 [M+Na]^+^ (calcd for C_11_H_14_O_3_Na, 217.0835).

*Pestalafuranone B* (**2**): pale yellow oil; [*α*]_D_ −16 (*c* 0.05, CH_3_OH); UV (CH_3_OH) λ_max_ 270 (*ε* 3300) nm; IR (Neat) *ν*_max_ 3403 (br), 2932, 1749, 1446 cm^−1^; ^1^H- and ^13^C-NMR data, see Table 1 and Table 2; HRESIMS obsd. *m/z* 217.0842 [M+Na]^+^ (calcd for C_11_H_14_O_3_Na, 217.0835).

*Pestalafuranone C* (**3**): pale yellow oil; UV (CH_3_OH) λ_max_ 265 (*ε* 6600), 235 (*ε* 4300) nm; IR (Neat) *ν*_max_ 2919, 1752, 1717, 1445, 1365 cm^−1^; ^1^H- and ^13^C-NMR data, see Table 1 and Table 2; HRESIMS obsd. *m/z* 219.0998 [M+Na]^+^ (calcd for C_11_H_16_O_3_Na, 219.0992).

*Pestalafuranone D* (**4**): colorless oil; [*α*]_D_ +32 (*c* 0.05, CH_3_OH); UV (CH_3_OH) λ_max_ 275 (*ε* 4460) nm; IR (Neat) *ν*_max_ 2963, 1751, 1450, 1381 cm^−1^; ^1^H- and ^13^C-NMR data, see Table 1 and Table 2; HRESIMS obsd. *m/z* 217.0846 [M+Na]^+^ (calcd for C_11_H_14_O_3_Na, 217.0835).

*Pestalafuranone D* (**5**): colorless oil; [*α*]_D_ +16 (*c* 0.05, CH_3_OH); UV (CH_3_OH) λ_max_ 275 (*ε* 6270) nm; IR (Neat) *ν*_max_ 2931, 1753, 1446, 1346 cm^−1^; ^1^H- and ^13^C-NMR data, see Table 1 and Table 2; HRESIMS obsd. *m/z* 219.0995 [M+Na]^+^ (calcd for C_11_H_16_O_3_Na, 219.0992).

### 3.4. Anti-HIV Assay

Anti-HIV assays were conducted in triplicate by the following methods: the inhibition of HIV replication, represented by the reduction of p24 antigen was determined by p24 antigen capture ELISA [8]. C8166 cells were exposed to HIV-1_LAI_ (MOI = 0.058) at 37 °C for 1 h, washed with PBS to remove free viruses, and then seeded into a 96-well microtitre plate at 3 × 10^4^ cells per well in the absence or presence of test compounds. AZT and IDV (provided by the NIH AIDS Reagent Program) were used as positive controls. After 4 days, the supernatant was collected and inactivated by 0.5% Triton X-100. The supernatant was diluted three times, added to the plate coating with anti-p24 McAb (provided by Dr. Bin Yan), and incubated at 37 °C for 1 h. After washing 5 times with PBST, the HRP labeled anti-p24 antibody (provided by Dr. Bin Yan) was added and incubated at 37 °C for 1 h. The plate was washed 5 times with PBST, followed by adding OPD reaction mixture and termination buffer. The assay plate was read at 490 nm using a microplate reader within 30 min. The inhibition rate and the EC_50_ based on p24 antigen expression level were calculated. 

### 3.5. Antifungal Bioassay

Antifungal bioassays were conducted in triplicate by following Clinical and Laboratory Standards Institute (CLSI) recommendations [9]. The plant pathogenic fungal strains were obtained from China General Microbial Culture Collection (CGMCC), and grown on potato dextrose agar (PDA). The fungal inocula were then prepared from broth cultures that were incubated at 25 °C for 48 h, and the final suspensions contained 10^4^ hyphae/mL of corresponding plant pathogenic fungi. Test samples (10 mg/mL as stock solution in DMSO and serial dilutions) were transferred to 96-well clear plate in triplicate, and the suspensions of test organisms were added to each well achieving a final volume of 200 μL (fluconazole was used as positive control; 10 μL of 10% alamar blue solution was added to each well as indicator). After incubation (28 °C for 48 h), the fluorescence intensity was measured at Ex/Em = 544/590 nm. The inhibition rate was calculated and plotted *versus* test concentrations to afford the IC_50_ values. 

## 4. Conclusions

This was the first report in which five new furanones pestalafuranones A–E (compounds **1**–**5**), were characterized from the endophytic fungus *P. besseyi*, which would further provide the evidence that endophytes, inhabiting a unique environment, could produce diverse secondary metabolites with a wide range of bioactivities.
